# Polish Nurses’ Knowledge of Heart Failure Self-Management Principles

**DOI:** 10.3390/ijerph19031327

**Published:** 2022-01-25

**Authors:** Dorota Krówczyńska, Beata Jankowska-Polańska

**Affiliations:** 1 The Cardinal Stefan Wyszynski Institute of Cardiology, Alpejska 42, 04-628 Warsaw, Poland; dkrowczynska@o2.pl; 2 The Center for Research and Innovation, 4th Military Teaching Hospital, Weigla 5, 53-114 Wroclaw, Poland

**Keywords:** coordinated care program, heart failure, nurses, knowledge, attitude

## Abstract

Background: Only comprehensive care and structured education can have a significant impact on the effectiveness of treatment and prepare patients for self-care. Unfortunately, Polish nurses are not ready to perform comprehensive heart failure (HF) care tasks without careful preparation.The purpose of the study was to evaluate nurses’ knowledge of patient education in general and topic-specific perceptions of basic information important for HF self-care, and also to determine the variables (workplace, education, internship) that may affect the implementation of educational tasks in the care of patients with HF. Methods: The study involved 304 nurses who were surveyed using the Nurses’ Knowledge of Heart Failure Education Principles. Results: Cardiology nurses’ knowledge regarding patient education for self-care is insufficient. The overall knowledge score was the highest among nurses in provincial specialist hospitals and university hospitals and the lowest in regional hospitals in small towns (14.98 vs. 14.35 vs. 12.83 vs. 11.89, respectively). Nurses who completed a cardiological specialty demonstrated significantly more extensive knowledge than those who had completed other specialties (15.52 vs. 13.71). Conclusions: Cardiology nurses’ knowledge of HF self-care principles regarding patient education is satisfactory, but not with all mandatory issues, especially in the recognition of disease symptoms, exacerbations, and pharmacotherapy.

## 1. Background

Heart failure is a worldwide problem in the 21st century, with an increasing effect on healthcare systems [1]. A significant increase in incidents is now being observed, especially among senior citizens. According to U.S. data, about 400,000 new cases are diagnosed each year [2]. Heart failure affects over 5.7 million individuals in the United States, and 15 million people in Europe. In Poland, the number of patients with HF has reached nearly 750,000, which means that HF is the third most common cardiovascular disease [2,3]. Heart failure is connected with a very high level of disease symptom exacerbation, disability and lack of patient activity, and low life quality, and it constitutes a burden and costs for the healthcare system [4]. 

Quality healthcare ensured for a patient suffering from heart failure and the challenges connected with it are issues for medical professionals. Frequent hospitalizations are due to the lack of organized outpatient care and one-day care for these patients. Moreover, shortcomings in pre-hospital care and lack of proper supervision, including lack of therapy optimization, are among the most common reasons for rehospitalization. Lack of treatment coordination leads to tests being performed twice, as well as to conflicting therapeutic and diagnostic decisions [5,6].

Due to the above-mentioned problems and difficulties in treatment for patients with heart failure, many countries have made efforts to organize efficient treatment and preventative heart failure programs, as well as comprehensive coordinated care aimed primarily at reducing the mortality rate, improving life quality for patients and their families, ensuring active supervision throughout the course of the disease, and limiting the number of hospitalizations, in addition to better use of the funds available for patient treatment [7].

The aim of commonly proposed coordinated care is to combine outpatient and inpatient care in order to provide the best care. 

In the assumptions of coordinated care, the patient is cared for by a multidisciplinary team with the crucial role of a qualified nurse acting as the leader. Most often, a nurse provides direct care for patients and his/her educational tasks are performed in both the hospital and home environment. The nurse performs the activities of the entire team and monitors the course of the established therapeutic plan. A pilot program proposed and implemented in Poland combines elements of outpatient care, pharmacotherapy, emergency medication, and rehabilitation. The proposed KONS program is another model of comprehensive care implemented by the Polish Ministry of Health and the National Health Fundafter the successful model of comprehensive care for patients after myocardial infarction, denoted by the acronym KOS [5]. The primary goal of the project KONS was to reduce the impact of HF in Poland. The scope of intervention begins with the identification of heart failure symptoms through diagnosis, therapy, rehabilitation, and long-term care and palliative care [7].

The inability of patients with HF to self-care and the low level of complying with therapeutic recommendations leads to poor HF treatment effectiveness [6]. Patients are unaware of the necessity of continuous treatment and monitoring symptoms, as well as diagnosing factors leading to exacerbation of the disease [8]. Based on everyday self-observation and monitoring of clinical parameters, there is a possibility to limit the risk of de-compensation. Unfortunately, only every second patient takes their medication regularly and a mere 10–15% of patients comply with all the therapy recommendations [6]. Engaging in physical activity and having a low-salt diet are the least observed recommendations [6,9].

Nurses play the role of educators in the currently implemented comprehensive care program for patients with heart failure [7].

The main education issues are as follows: monitoring symptoms, regular administration of medications, dietary treatment, managing and recognizing exacerbation, monitoring weight, diet, level of activity, medication, and follow-up appointments [9].

The effectiveness of education in the self-care preparation of patients depends on the patient’s abilities and attitude, but also on the skills of the nurse to carry out these tasks [10]. Unfortunately, previous studies indicated an insufficient level of knowledge among Polish nurses to perform comprehensive HF care tasks as educators [11].

There is little research devoted to assessing the preparedness of nurses to act as health educators. Systematized insights into Polish nurses’ knowledge of HF self-care education principles might identify gaps that can be filled by introducing appropriate training during vocational education or supplementary education during postgraduate training.

Theoretical framework to Understand heart failure self-care principles.

**Figure 1:**
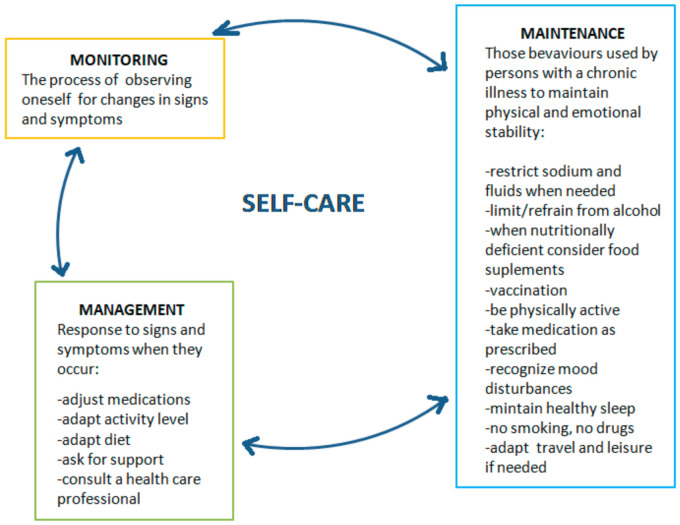
Theory of self-care in heart failure.

Self-care is essential in the management of chronic illness (Figure 1). The process of self-care consists of three essential elements of self-care maintenance, self-care monitoring, and self-care management. These elements are interrelated and cannot be considered as separate in the patient education process. The essence of preparing a patient with a chronic disease for self-care is to master these three elements in sequence [12].

Self-management is defined as one’s control over and responsibility for the management of chronic conditions or healthy behaviors by purposefully engaging in the performance of learned behavior [13]. The goldstandard of patient self-management in chronic heart failure (CHF) can be defined as “daily activities that maintain clinical stability” [14].

This requires patients to monitor symptoms; adhere to medication, diet, and exercise regimens; and manage symptoms by recognizing changes and responding by adapting behaviors or seeking appropriate help [13]. According to the European Society of Cardiology Guidelines for the diagnosis and treatment of acute and chronic heart failure, self-management is integral to achieving the best patient outcomes: to reduce mortality and improve quality of life [15]. Self-management in CHF usually involves behavioral adaptation. Patients should learn to monitor their coping with symptoms and complex treatment regimens. Patients must have the knowledge and skills to perform self-management techniques to prevent or reduce negative consequences of HF and optimize their quality of life.

Studies show that education during hospitalization and ambulatory education programs improve clinical outcomes of HF treatment, reduce readmission rates, and reduce health care costs [16,17,18,19].In many countries, the tasks of educators revolve around nurses, who must be able to assess patients’ knowledge deficits and have the knowledge to deliver educational interventions. Unfortunately, research shows that nurses may not have the appropriate knowledge of HF management to educate patients and consequently are not optimally equipped to provide appropriate HF education [20,21,22].

## 2. Methods 

### 2.1. Study Design and Sample

This was a cross-sectional study performed in a public and university hospital setting with the use of a descriptive design and survey method. The study included nurses working in units where HF patients were hospitalized: the non-invasive treatment ward, one-day procedures ward, telemetric monitoring cardiology ward, intensive cardiology care ward, cardiosurgery ward, and cardiology rehabilitation ward (those which provide care to HF patients) [11].

Nurses who are involved in the care of patients with heart failure in their work were invited to participate in the study. 

Inclusion criteria: part- and full-time registered nurses with medical secondary school education, bachelor’s degree or master’s degree, professional duties involving HF patient care, consent to participate in the study [11].

Exclusion criteria: no earlier experience in care of patients with heart failure, no willingness to participate in the study [11].

The information about the study was posted on a social networking portal for nurses, along with the invitation to participate. A total of 657 nurses agreed to participate in the study; however, due to the criteria set, 155 were not qualified. 

Although an invitation to participate in the study was sent to 503 nurses employed mainly in cardiac units, only 304 accepted. The final number of 304 nurses were asked to fill two questionnaires on Nurses’ Knowledge of Heart Failure Education Principles [10]. The nurses who failed to complete the questionnaire did not give any reason for their refusal to do so. All nurses included in the study received information about the purpose of the study, its voluntary nature, and its risks and benefits. They were also informed that participation in the study may increase their knowledge of HF. Informed written consent was obtained from all participating individuals. The nurses participating in the tests completed the questionnaires on their own and at a time they chose. The study was carried out in accordance with the tenets of the Declaration of Helsinki. The study was approved by the Bioethics Committee of the Wroclaw Medical University (KB 205/2019).

### 2.2. Instrument

The Nurses’ Knowledge of Heart Failure Education Principles survey was used with the consent of its author, Albert Nancy. The questionnaire has already been used in Polish studies for which the translation procedure was completed using internationally accepted standards [23]. The questionnaire consists of 20 questions to which the answers “yes”, “no”, or “don’t know” are given. The questions included in the questionnaire are arranged in 5 domains relating to the most important educational aspects of HF self-maintenance: diets (3 statements); weight and fluids (7 statements); signs or symptom of worsening condition (6 statements); medications (2 statements); and exercise (2 statements). When filling in the survey, it was possible for the respondents to mark questions for which they needed more information. Answers “don’t know” in the survey were considered irrelevant.

In the Nurses’ Knowledge of Heart Failure Education Principles survey, there is no generally accepted score, which is why we applied the following standards:

- 0–10 of correct answers (0–50% questions): inadequate knowledge;
- 11–15 of correct answers (50–75% questions): satisfactory knowledge;
- 16–18 of correct answers (75–90% questions): good knowledge; and
- 19–20 of correct answers (90–100% questions): very good knowledge.

Additionally, we collected information on the participants’ characteristics (age, gender, education, postgraduate training) and workplace (type of hospital: (1) big city hospital, (2) regional hospital in a small town, (3) multidisciplinary provincial specialist hospital, (4) university hospital, and (5) private sector). We also obtained information on the type of ward.

### 2.3. Statistical Analysis

The sums of correct responses to individual items (total correct response) were calculated and described as frequencies, means, and standard deviations.

Comparisons of the quantitative variables were conducted using the Mann–Whitney test. Comparisons of quantitative variables in more than two groups were conducted with the Kruskal–Wallis test. Dunn’s test was used as a post hoc procedure. Correlations between quantitative variables were assessed with Spearman’s correlation coefficient. Analyses were conducted at the 0.05 level of significance. The significance level was set at 0.05 to show that the conditional probability of a type I error, given that the null hypothesis is true, is 5%.

## 3. Results

A total of 304 nurses (age average 42.55 ± 10.03) participated in the survey. They work in wards caring for patients with heart failure. 

Over 70% of survey respondents had a university degree, but only every fourth nurse had completed specialist training (25.33%). The detailed characteristics of the study group are presented in Table 1.

### 3.1. Evaluation of Preparing for Education and Knowledge Regarding Issues of HF Patients and Their Preparation for Self-Care (The Nurses’ Knowledge of Heart Failure Education Principles Questionnaire)

**Figure 2:**
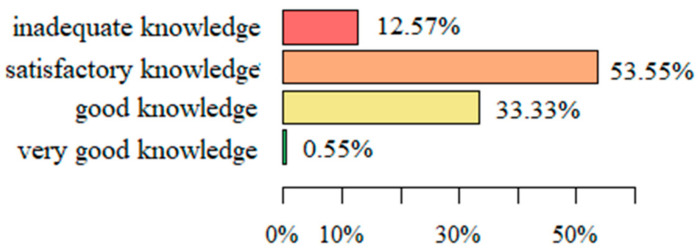
The level of knowledge regarding issues of HF patients and their preparation for self-care.

In the survey, 98 of 183 respondents who filled in the survey (53.55%) demonstrated satisfactory knowledge, 61 (33.33%) demonstrated good knowledge, and 23 (12.57%) demonstrated inadequate knowledge. Only one respondent obtained a very good result (0.55%) (Figure 2). 

The average number of correct answers (mean ± SD) in the entire survey was 13.94 ± 2.78 and was in the range of 6 to 19. This means that none of the respondents gave correct answers to all questions in the questionnaire and the obtained result confirms the average skills of nurses in carrying out educational tasks preparing patients for self-care. 

### 3.2. Characteristics of Answers Regarding the Specific Subject Areas

**Figure 3:**
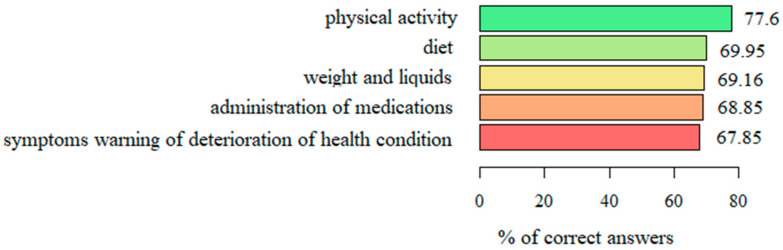
The level of knowledge regarding the specific subject areas.

The respondents demonstrated the best knowledge regarding questions about physical activity, as 77.6% on average gave correct answers. Regarding the other domains of the questionnaire, similar scores were achieved for diet (69.95%), weight and liquids (69.16%), and administration of medications (68.85%). The lowest percentage of correct answers was given with respect to symptoms warning of a deterioration in health condition (67.85%) (Figure 3).

### 3.3. Knowledge Regarding Individual Questions in the Nurses’ Knowledge of Heart Failure Education Principles Questionnaire

The questions that received the highest number of correct answers were the question regarding the role of lean meat in the diet of HF patients (96.72%), the question related to the symptoms of a deterioration in leg motion and reduced ability to perform exercises (93.99%), and the question related to liquid build-up in the abdominal cavity as a symptom of worsening HF (91.26%).

The most difficult questions, which received the lowest number of correct answers, were the question related to the evaluation of everyday body weight and referring to its value as the “dry body weight” (20.77%); the question related to diagnosing symptoms indicating HF diagnosis, i.e., loss of appetite, cough, and nausea (37.16%); as well as replacing salt in everyday diet (41.53%). The responses to all questions in the survey are presented in Table 2.

### 3.4. Analysis of Correlations between the Selected Sociodemographic Variables and the Level of Knowledge Related to the Score in The Nurses’ Knowledge of Heart Failure Education Principles Questionnaire

The results of the analysis show that neither age nor professional experience correlated with the level of knowledge related to the nurses’ preparation for educating patients for self-care. However, a correlation was observed between the type of hospital where nurses were employed and the level of knowledge. Nurses in provincial specialist hospitals (14.98 ± 1.94) and university hospitals (14.35 ± 2.85) demonstrated significantly more extensive knowledge than the respondents (12.83 ± 2.88) from city hospitals and (11.89 ± 3.46) regional hospitals in small towns. Furthermore, it was observed that staff in cardiological rehabilitation wards (15 ± 3.1), cardiosurgery (15.43 ± 1.8), and intensive cardiology care wards (14.68 ± 2.11) demonstrated significantly more extensive knowledge than staff in one-day procedure wards (12.53 ± 2.78) and in invasive cardiological care (13.54 ± 1.9). Nurses working in shifts (day, night) demonstrated more extensive knowledge than nurses working only in the morning shift ((14.47 ± 2.79) vs. (13.33 ± 2.55)). The analysis of correlations between education and knowledge showed that nurses with a higher level of education had significantly more extensive knowledge (14.24 ± 2.78) than those with a secondary level of education (13.3 ± 2.68). Notably, differences occurred among nurses with specialties and those without ((14.7 ± 2.36) vs. (13.39 ± 2.94)). When comparing the effectiveness of two-year specialist training, it should be emphasized that nurses who completed a cardiology specialty demonstrated significantly more extensive knowledge than those who had completed specialties in other fields of nursing ((15.52 ± 1.65) vs. (13.71 ± 2.84)) (Table 3).

## 4. Discussion

Heart failure is the leading cause of death related to cardiovascular disease. A major problem for patients with HF is repeated hospitalizations due to exacerbations of the disease. Numerous rehospitalizations are associated with a lack of continuity of care after hospital discharge, both specialty and primary care. The system lacks proper education directed at patients with heart failure, aiming at preparing patients for self-care in terms of maintenance, management, and monitoring. Multi-specialist care provided by a team is important in the treatment of HF patients. Such multi-specialist care must define very clearly the crucial role of qualified nurses in the care of HF patients. In Poland, action has been undertaken to implement the KONS program, combining all levels of care of HF patients. A very important part of this is the patient education program conducted by prepared educators, especially nurses who are trainers. The purpose of this education is to improve the quality of cooperation between patients and medical staff, and the patient’s ability to self-estimate and self-care [7].

Our study shows that with respect to important areas of patient education, nurses are not adequately prepared for situations when they should be an important source of information for the patient.

The available publications show inadequate preparation of nurses for the role of cardiology educators [6,10,24,25]. According to our own study, nurses’overall preparation for the role of educator and educating a patient before their discharge from hospital was evaluated at 13.9. Over half of respondents showed only a satisfactory or inadequate level of preparation for performing the role of educators. The results obtained for Polish nurses are comparable to those for nurses in Cyprus, 13.57 (67.8%) [24], and are lower than the results published by authors describing U.S. surveys [23].

In our present study, a slight improvement in knowledge among Polish nurses was observed compared to the results obtained in Poland in 2017 [8]. However, the results of the study may still raise concerns that the percentage of nurses with an inadequate level of preparation for educating patients is so significant that it can interfere with the education activity and fail to sufficiently prepare patients to participate consciously in the therapeutic process. 

In a previously published Polish study, the low level of knowledge among Polish nurses could have resulted from several reasons, including age, no university education, and no specialist postgraduate courses being available for educating cardiology nurses [6].

Although the current group of nurses under survey was slightly younger than the group examined in 2017, the average age of a cardiology nurse is over 40 and the average professional experience is around 20 years. 

In our present study, the majority of nurses were educated at the academic level, but as was the case before, many did not undertake further education at the level of postgraduate specialty training.

It is worth noting that higher results obtained in the current study might have the following causes: first, this group mostly comprises nurses employed at large, multidisciplinary provincial specialist hospitals and university hospitals. An analysis of correlations between the selected sociodemographic variables and the level of knowledge showed that nurses in provincial and university hospitals demonstrated significantly more extensive knowledge than other respondents [6]. A detailed comparative analysis of the level of preparation depending on selected variables gave rise to a definite statement that having a specialty was a factor improving the preparation of nurses for educating patients, and such nurses were even better evaluated if their specialization was connected with cardiology. The results of our study are consistent with surveys by other authors, in which the knowledge of nurses who did not have additional preparation as part of specialty training was evaluated as the lowest [6]; in addition, differences were observed among nurses who had only completed a specialist course and those who had finished two-year specialist training. The authors emphasize that all training and interventions undertaken by nurses for increasing their knowledge and competence have a positive effect on their knowledge of HF education [26]. Similarly, in our own study, better results were achieved by nurses with a university education in comparison to those without such a level of education. In another study evaluating the relationship between the level of education and the comfort and frequency of educating patients with heart failure by nurses, it was shown that the level of education had a significant impact on both the comfort and the frequency of educational activities [11]. The authors emphasized that nurses with a cardiological specialty educated patients more often and experienced more comfort during educational actions compared to nurses who had completed other specialty training [11].

What is interesting is that the available studies contain contradictory information regarding a connection between professional experience and the level of preparation for educating HF patients [24,25,26,27]. In our study, no relationship between experience and education was found, but in a previously published study [6], a longer job experience was a factor negatively contributing to the level of a nurse’s preparation for educating HF patients. However, in the study conducted by Krówczyńska, professional experience exerted a statistically important influence on nurses’ comfort with providing education for HF patients but did not affect the frequency of this education [11].

Moreover, the results of our own research, which emphasize the relationship between the level of education and the preparation of nurses for educating HF patients, are in disagreement with those previously published [27,28]. What is interesting to note is that in Krówczyńska’s study, nurses working in regional hospitals in small towns experienced the most comfort and frequency in educating HF patients [11]. Unfortunately, it is difficult to explain the situation and further research on that group of nurses should be conducted. Perhaps it is the case that in such hospitals, education to prepare patients for self-care is conducted most often, but the staff have no access to postgraduate training and university studies to the same degree as staff from provincial and university hospitals. Moreover, even if education is provided, it is not sufficient, or not to the extent that patients expect. These results suggest that nurses working in small community hospitals may not be sufficiently knowledgeable of HF management principles [6,8].

## 5. Conclusions

Cardiology nurses’ knowledge of HF self-care principles regarding patient education is satisfactory, but not with all mandatory issues. Nurses were not sufficiently prepared to provide patients with education on HF self-care principles related to recognizing warning symptoms and deteriorations in health conditions and in the administration of medications.

A higher level of education and postgraduate education, especially cardiological specialties, are determinants that have a significant impact on the level of Polish nurses’ knowledge of HF self-care principles, which results in greater effectiveness in performing the task of educating patients with HF.

Firstly, having adequate knowledge in the field of self-management principles will facilitate targeted education on the basis of the individualized needs of patients. 

Furthermore, nurses who know the principles of HF self-care can better prepare for work and education as part of an interdisciplinary team.

In conclusion, the identified areas of knowledge deficits among Polish nurses regarding HF self-care principles may lead to problems in providing patients with an adequate level of self-care education.

### 5.1. Study Implications

It is important to critically examine the gaps in knowledge, particularly in the areas of asymptomatic hypotension, transient dizziness, and weight monitoring. There is a need to develop interventions to improve nursing knowledge of HF self-management principles. Clinical nurse specialists can be instrumental in developing knowledge interventions for nurses.

### 5.2. Summary

A nurse is a team member who plays the most important role in the objective monitoring of the patient’s condition, as well as participating in coordinating hospital care, planning timely intervention after discharge from the hospital, engaging a patient and/or their family in self-care, and striving for cooperation efficacy and communication with the treatment team. Unfortunately, all this is not possible without relevant prior preparation, training, and acquiring the skills of a teacher and educator. At this stage of implementing the project, it is important to provide nurses with the subject matter care and professional knowledge so that they can become conscious and professional members of a team and carry out the tasks of medical educators. 

### 5.3. Study Limitation

Our research has some limitations. One of them is the selection of nurses, which did not allow for the creation of equal groups. This indicates a need for the continuation of research with reasonable, thoughtful recruitment of nurses. It is necessary to consider the discrepancies between the territorial location of facilities and the size and type of hospital, so that individual groups are similar in size and comparable.

Another study limitation is the lack of information about the on-the-job training systems and the employment system (qualification requirements, experience) in a given position of a nurse who performs the role of an educator, among other tasks.

In future research, it is worth evaluating the system of internal training, which can prove helpful in preparing nurses starting their professional career, and the role of the nurse specialist as an advisor and a person supervising educational activities.

## Figures and Tables

**Table 1:** Group characteristics of the nurses participating in the study.

Characteristic		Values
Age	Mean (SD)	42.55 (10.03)
	Me	45
Years of experience	Mean (SD)	19.41 (11.34)
	Me	21
Site	City hospital	36 (11.84%)
	Regional hospital in small towns	19 (6.25%)
	Provincial specialist hospital	87 (28.62%)
	University hospital	161 (52.96%)
	Private sector	1 (0.33%)
Gender	Female	276 (90.79%)
	Male	28 (9.21%)
Place of work	Non-invasive treatment ward/Intensive care unit	45 (14.80%)
	Emergency care ward/Short-term care	36 (11.84%)
	Telemetric monitoring cardiology ward	89 (29.28%)
	Intensive cardiology care ward	77 (25.33%)
	Cardiosurgery ward	21 (6.91%)
	Cardiology rehabilitation ward	17 (5.59%)
	Outpatient care	19 (6.26%)
Work schedule	Usually a day shift, on weekdays	88 (28.95%)
	Usually other than a day shift, on weekdays	27 (8.88%)
	Usually weekend shifts	1 (0.33%)
	Balance of day and other shifts	180 (59.21%)
	Temporary/irregular work	8 (2.63%)
Level of education	Secondary	88 (28.95%)
	University	216 (71.05%)
Specializations	Yes	77 (25.33%)
	No	227 (74.67%)

SD—standard deviation, Me—median.

**Table 2:** Percentage of correct answers to all questions from the Nurses’ Knowledge of Heart Failure Education Principles survey (best results).

Question	% of Correct Answers
13. Lean delicate meat can be a part of the diet of patients with heart failure	96.72
20. New onset or worsening of leg weakness, or lower ability to do exercises	93.99
6. Abdominal swelling may suggest that excessive fluid is retained due to worsening heart failure	91.26
19. New onset or worsening of fatigue	89.62
1. Patients with heart failure should drink a lot of fluids every day	86.89
14. Once patients’ heart failure symptoms are gone, there is no need for them to obtain daily weights	83.06
17. A 2 kg weight gain in 5 days without symptoms	82.51
7. If patients take drugs and modify their lifestyle as recommended, their heart failure will not recur	78.14
16. BP of 80/56 without any heart failure symptoms	77.05
2. As long as food is not salted, there are no dietary restrictions for patients with heart failure	71.58
5. If patients put on weight more than 2 kg in 48 h without other heart failure	69.95
12. If patients wake up at night and have breathing problems, and if these problems abate when patients get out of bed and walk around, it does not mean that heart failure has worsened	69.4
18. Dizziness when getting up which subsides within 10–15 min	69.4
11. When patients use additional pillows at night to relieve short breath, it does not mean that heart failure has worsened	64.48
4. Patients with heart failure should limit activity and avoid most forms of exercises	61.2
8. When patients feel pain, aspirin and non-steroid anti-inflammatory drugs (such as ibuprofen) should be recommended	59.56
10. If patients feel thirsty, they can be allowed to give up the fluid regimen and drink	49.73
9. Potassium-based salt substitutes (ex. “no-salt” or “salt-sense”) can be used to season food	41.53
3. Coughing and nausea/loss of appetite are common symptoms of advanced HF.	37.16
15. When assessing weight results, patients’ weight on a given day should be compared to their weight on the previous day, and not their ideal or ‘dry’ weight	20.77

HF—heart failure.

**Table 3:** Analysis of correlations between selected sociodemographic variables and the level of knowledge related to score of the Nurses’ Knowledge of Heart Failure Education Principles questionnaire.

	Parameter	Knowledge Level	*p* Value
Type of hospital	City hospital	12.83 ± 2.88	<0.001 NP
	Regional hospital in small town	11.89 ± 3.46	
	Provincial specialist hospital	14.98 ± 1.94	
	University hospital	14.35 ± 2.85	
Ward	Non-invasive treatment ward/Intensive care unit	13.54 ± 1.90	0.001 NP
	Emergency care ward/Short-term care	12.53 ± 2.78	
	Telemetric monitoring cardiology	13.69 ± 3.00	
	Intensive cardiology care	14.68 ± 2.10	
	Cardiosurgery	15.44 ± 1.80	
	Cardiology rehabilitation	15.00 ± 3.10	
Type of job	Usually dayshift, on weekdays	13.33 ± 2.55	0.013 NP
	Usually other shiftthan on weekdays	13.72 ± 2.44	
	Balance of dayand other shifts	14.47 ± 2.79	
Level of education	Secondary	13.3 ± 2.68	0.038
	Higher	14.24 ± 2.78	
Postgraduate education	Specialty	14.7 ± 2.36	0.004
	No specialty	13.39 ± 2.94	
Type of specializationeducation	Cardiological specialty	15.52 ± 1.65	0.002
	Specialtyother than cardiological	13.71 ± 2.84	
Correlation with the level of knowledge			
**Parameter**		**Correlation rate**	***p* value**
Age		−0.088	*p* = 0.244 NP
Professional experience		−0.062	*p* = 0.401 NP

*p* = normal distribution among groups, NP = no normal distribution among groups, Mann–Whitney test.

## Data Availability

The data are not publicly available due to privacy and ethical restrictions. The data presented in this study may be available conditionally from the corresponding author.
